# The criteria used by key decision makers in Australia to judge the academic quality of NTROs

**DOI:** 10.1177/1329878X20921565

**Published:** 2020-11

**Authors:** Alan McKee

**Affiliations:** University of Technology Sydney, Australia

**Keywords:** Excellence in Research for Australia, higher education policy, research assessment, research policy, research quality

## Abstract

Thirty experts in the assessment of the quality of Non-Traditional Research Outputs (NTROs) as academic research outputs were asked to rate the importance of 19 criteria that might be used in making these judgements. Analysis of responses identified four criteria where there is substantial agreement among the community of experts: (a) demonstrated familiarity in the research statement with the current state of knowledge in the relevant academic disciplines (very important); (b) demonstrated familiarity in the research statement with the current state of knowledge in the relevant industry (important); (c) evidence that the work has been engaged with by other academic researchers (relevant); (d) whether the NTRO creator is a substantive university staff member or an adjunct/honorary (unimportant). Fifteen other criteria either reached a less than ‘fair’ level of agreement, or larger numbers of respondents nominated ‘It depends’. Qualitative analysis of comments also revealed noteworthy disagreements in the expert community about how the criteria should be applied.

## Introduction

Many countries now have national systems established to evaluate the quality of academic research, each of which functions slightly differently (Rebora and Turri, 2013). Although there is relatively little academic research conducted on these systems, it is important that we subject them to the same level of academic scrutiny as other aspects of research policy – as for example Smith et al. (2011) do in their consideration of how impact is evaluated in the UK Research Excellence Framework. This article addresses a problematic aspect of the Australian ‘Excellence in Research for Australia’ (ERA) research evaluation system. This system includes a category of outputs named as ‘Non-Traditional Research Outputs’ (NTROs), which encompasses creative works, public exhibitions and events, or research reports produced for an external body, or portfolios of such works, that meets the Australian Research Council’s (2017) definition of academic research:

the creation of new knowledge and/or the use of existing knowledge in a new and creative way to generate new concepts, methodologies, inventions and understandings. This [can] include the synthesis and analysis of previous research to the extent that it is new and creative. (p. 9)

NTROs have been an established part of the Excellence for Research in Australia evaluation system since the pilot assessment run in 2010. However, looking at the average scores in each ERA round suggests a systemic problem. The average scores of most Fields of Research have increased in each subsequent round of ERA (which were run in 2012, 2015 and 2018) as institutions have understood and responded to the understanding of research quality that ERA intends to measure and promote. But those FoRs which rely most strongly on NTROs – in 12 and 19 – have not improved, and in some cases have actually decreased. FoRs 12 and 19 are the codes which present most NTROs for analysis. It might be hypothesised that the failure of these codes to follow the trend of other codes could be related to a lack of a clear, national, sector-wide understanding of the ways in which assessors are conceptualising the academic quality of NTROs.

There exists a significant and robust academic literature on practice-led and practice-based research (see, for example, Borgdoff, 2012; Candy, 2006; Coessens et al., 2010; Freeman, 2012; Haseman, 2006; Nelson, 2013; Smith and Dean, 2009). Indeed, Australia is home to *NiTRO*:

a platform for creative artists practicing in academia to contribute to informed discussion about issues and activities relating to practice, research and teaching taking place within the university sector. (https://nitro.edu.au/about)

These publications have developed a robust epistemological position from which to argue for the nature of creative practice research in academia, and the particular ways in which it might function, as well as arguing passionately for its importance. However, we have less systematic knowledge about the operationalisation of the evaluation of practice-led and practice-based research at the national level, through the evaluation of NTROs in ERA (and also in other arenas, such as the assessment of grant applications by the Australian Research Council).

The data presented in this article emerge from a project conducted for the Australasian Council of Deans of Arts, Social Sciences and Humanities (DASSH). DASSH is ‘the authoritative agency on research, teaching and learning for the Arts, Social Sciences and Humanities (ASSH) in Australian and New Zealand universities’ (https://dassh.edu.au/). At the meetings of DASSH Associate Deans Research, NTROs are a regular topic of discussion. It became clear in these discussions that although the group had consistently interesting and exciting conversations about NTROs, we did not have a clear idea about how key decision makers involved in scoring ERA submissions, assessing ARC applications and other forms of research assessment at the national level are actually judging the quality of NTROs as academic research (as opposed to their quality as works of art, for example). Given that the members of this group are responsible for responding to ERA and developing culture and practice in Australian institutions to develop research quality in NTROs, this was a problem. For this reason, DASSH supported a research project to contact key decision makers in Australia and survey them about the criteria they used to make decisions about the quality of NTROs as academic research. This article reports on the findings from this project. It was envisaged that these data will be useful for academics who are involved in research leadership at all levels, including Associate Deans Research as well as those working in and with the Australian Research Council. It will also be useful for research policy makers, and for academics who are interested in research policy and cultural policy. It will also be of interest to academics who produce NTROs – not so much for thinking about their own practice, but in terms of better understanding the sectoral landscape within which they are working.

Much of the writing on NTROs has taken a prescriptive tone – this is how things *should* be done. By contrast, this project seeks to take a descriptive approach – this is how things *are* being done. For this reason, this article is not structured around an argument about what next steps must be taken in the NTRO space; beyond perhaps suggesting that as research policy makers consider next steps in managing the NTRO space, that it would be useful to refer to these data about how assessment of NTROs is currently being conducted.

## Method

As a first step in the process an Advisory Group was established involving representatives from key stakeholder organisations, with a particular focus on those that hold a thought-leader status in regard to the development of NTROs in Australia. The following organisations were involved in the development of the project:

- Australian Research Council (observer status)
- Australian Council of Deans and Directors of Creative Arts
- Australian Deans of Built Environment and Design
- Australian Council of University Art and Design Schools
- Council for the Humanities, Arts and Social Sciences
- Australian Academy of the Humanities
- Australian Screen Production, Education and Research Association
- Journalism Education and Research Association of Australia
- Australasian Association of Writing Programmes

The Advisory Group proposed names of suitable NTRO key decision makers at the national level and approved a process of asking these experts to recommend others who should be approached for the survey. Members also suggested the possible criteria that should be included – between them they generated 19 possible criteria that reviewers might use to evaluate the quality of NTROs as academic research. They then reviewed drafts of a proposed survey instrument. In total, 37 experts were proposed and contacted. Thirty of these key NTRO decision makers subsequently completed the survey (see Supplemental Appendix A).

The survey instrument first asked experts to nominate the FoR codes in which they have assessed the quality of NTROs as academic research. Relevant codes were identified by choosing the Fields of Research in ERA 2015 which submitted larger numbers of NTROs. The survey focused on creative outputs as research and datasets as research. We did not focus on reports, because the network of ADRs had not reported difficulties with understanding how reports were being assessed in ERA. We did ask a question about assessing datasets as research outputs, but too few respondents answered the question to allow us to report on this.

Respondents were also invited to nominate any other FoR outside the main list in which they had expertise. Respondents were then asked, ‘In assessing the quality of a NTRO as academic research, how important is each of the following criteria on a scale from Very unimportant, to Very important?’ and listed 19 possible criteria.

It is worth noting that the wording of this introductory question drew the attention of respondents to the fact that the survey was interested in the value of the NTROs as academic research and then asked them to consider other factors in relation to academic research. One of these factors was, ‘The success of the work according to the criteria of the practice community’. An ongoing debate in the NTRO community is whether these two qualities are different, or the same thing. It could be argued that by asking survey respondents to consider the relationship between these two factors the survey was already taking a side in this argument by implying that quality as academic research and the judgements of the practice community are different things. In response to this concern, I note that the survey asked respondents to specify the relationship between these terms and invited them to comment. Those survey respondents who believed that these were not separate factors, but the same factor, or strongly related, were able to make this point in their responses – as we will see in the analysis of qualitative comments, below.

It is also worth noting that we did not define for respondents what we meant by the ‘quality’ of academic research. As noted above, the aim of this project was to provide descriptive information about the ways in which those people who are currently making assessments about the quality of NTROs in academic contexts – for ERA, for ARC and so on – are currently making those decisions. All of these experts are currently employing notions of research quality in the assessments they are being engaged to do. It may be that they are all using it in different ways – this would make a fascinating follow-up project.

The design of the survey instrument drew on the early findings of a current research project that seeks to produce shared paradigms and vocabularies across research in the humanities and social sciences (DP170100808). This project has found that humanities researchers are less comfortable than social science researchers with 5-point Likert-type scales; conversely, humanities researchers are more likely to use an option to nominate ‘It depends’ and provide more extensive qualitative feedback in answer to a question. As the key NTRO decision makers in Australia tend to come from the humanities and creative arts rather than quantitative social sciences, the survey was designed using a 5-point Likert-type scale, but giving respondents the option to opt out of that scale for a given question by nominating ‘It depends’ and instead providing a qualitative response. Finally, respondents were invited to offer any further comments (complete survey instrument is attached as Supplemental Appendix B). Ethics approval for this project was granted through the UTS Ethics process, approval number UTS HREC ETH18-2746N.

## Results and analysis

The data were analysed to determine the criteria used in assessing the quality of NTROs as academic research where there is most agreement about their (un)importance. A mixed-method approach using two forms of analysis was employed.

First, the criteria were ranked according to the level of agreement among the experts about how important they are, based on a statistical analysis of results in order to determine the adjusted standard deviation for each criterion. In this analysis, the ‘It depends’ responses, which opted out of providing an answer on the Likert-type scale, were excluded and held over for analysis in the second, qualitative stage. The statistical analysis was conducted by Statistical Solutions (https://www.statisticalsolutions.com.au/). They were also asked to provide a layperson’s guide to the level of agreement represented by the standard deviations recorded: they provided the taxonomy Strong/Fair/Mild/Weak/None.

Second, criteria were ranked according to how many experts used the ‘It depends’ option to opt of scoring their importance, supported by a qualitative analysis of comments made in relation to ‘It depends’ answers to identify the discourses employed by key decision makers in relation to these criteria.

These two forms of analysis allowed for the identification of the criteria for assessing the quality of NTROs as academic research for which there is most agreement among the community of experts. Full details of the both steps in the analysis follow below.

The analysis showed that of 19 possible criteria nominated by the Advisory Group to assess the quality of NTROs as academic research outputs, there are four where there is general agreement among the community of experts as to their relative importance in assessing the quality of NTROs as academic research:

- Demonstrated familiarity in the research statement with the current state of knowledge in the relevant academic disciplines (very important);
- Demonstrated familiarity in the research statement with the current state of knowledge in the relevant industry (important);
- Evidence that the work has been engaged with by other academic researchers (relevant);
- Whether the NTRO creator is a substantive university staff member or an adjunct/honorary (unimportant).

In total, seven criteria had ‘Strong’ or ‘Fair’ levels of agreement among the experts surveyed in the first stage of analysis, excluding responses of ‘It depends’: Evidence that the work has been engaged with by other academic researchers (judged as being Relevant); Demonstrated familiarity in the research statement with the current state of knowledge in the relevant academic discipline(s) (very important); The success of the work according to the criteria of the practice community (important); Demonstrated familiarity in the research statement with the current state of knowledge in the relevant industry (important); Whether the NTRO creator is a substantive university staff member of an adjunct/honorary (unimportant); The scale of the NTRO (important); and Whether the NTRO has been critically reviewed in a journal, magazine or book (relevant).

However of these seven, three also had larger numbers of experts in the second stage of analysis nominating ‘It depends’ and providing comments that indicated divisions in the community of expert assessors: the scale of the NTRO (56%); the success of the work according to the criteria of the practice community (30%); and whether the NTRO has been critically reviewed in a journal, magazine or book (26%).

In addition to the analysis of the full cohort of respondents, a quantitative analysis was also undertaken of the responses in each FoR where respondents nominated an expertise (Supplemental Appendix C). This analysis should be treated with caution because of small numbers involved (in one case, for example, only two respondents were experts in the FoR) (Tables 1 and 2).

**Table 1.** Criteria by level of agreement about what is important.

	Standard deviation	Agreement, excluding ‘It depends’	How important is the criterion?
Evidence that the work has been engaged with by other academic researchers	0.88	Strong	Relevant
Demonstrated familiarity in the research statement with the current state of knowledge in the relevant academic discipline(s)	0.91	Strong	Very important
The success of the work according to the criteria of the practice community (i.e. whether it is a great piece of art, etc.)	0.98	Strong	Important
Demonstrated familiarity in the research statement with the current state of knowledge in the relevant industry	0.99	Strong	Important
Whether the NTRO creator is a substantive university staff member or an adjunct/honorary	1.06	Fair	Unimportant
The scale of the NTRO (e.g. would you score a portfolio of five pieces of work more highly than those five pieces submitted as individual NTROs? A feature film more highly than a short film?)	1.09	Fair	Important
Whether the NTRO has been critically reviewed in a journal, magazine or book	1.19	Fair	Relevant
Whether the work represents an advance on the present state of practice in the area	1.22	Mild	Important
Evidence of industry/community engagement	1.27	Mild	Important
Whether the work has been shortlisted for non-academic awards	1.30	Mild	Relevant
The presence of a strong academic research question in the research statement	1.39	Mild	Important
The standing as academic researchers of other creators whose outputs are presented in the same context (e.g. a group show of artists)	1.42	Mild	Relevant
The rigour of the programme of academic research as demonstrated in the research statement and/or the work itself	1.51	Weak	Important
Whether the work is practice-based, practice-led or uses practice to report on data generated through traditional research methods (such as a historical novel, for example)	1.68	Weak	Relevant
Whether the NTRO producer was the creator of the work, or a curator/editor of the work	1.80	Weak	Important
Whether the work is unique	1.86	None	
Evidence of academic peer review	1.93	None	
The standing of practitioners of other creators whose outputs are presented in the same context (e.g. a group show of artists)	1.96	None	
Whether a work is high culture or popular culture	2.47	None	

NTRO: Non-Traditional Research Output.

**Table 2.** Criteria by number of experts responding ‘It depends’.

	Percentage of experts responding ‘It depends’
Demonstrated familiarity in the research statement with the current state of knowledge in the relevant academic discipline	3
Whether the work is practice-based, practice-led or uses practice to report on data generated through a traditional research method (such as a historical novel, for example)	3
The rigour of the programme of academic research as demonstrated in the research statement and/or the work itself	6
Demonstrated familiarity in the research statement with the current state of knowledge in the relevant industry	10
Evidence that the work has been engaged with by other academic researchers (including invitations to present academic keynotes, etc.)	10
Whether the work has been shortlisted for non-academic awards	10
Evidence of industry/community engagement	10
The presence of a strong academic research question	13
The standing as practitioners of other creators whose outputs are presented in the same context (e.g. a group show of artists)	13
The standing as academic researchers of other creators whose outputs are presented in the same context (e.g. a group show of artists)	13
Whether the work is high culture or popular culture (e.g. a literary novel vs a Harlequin Mills and Boon)	13
Whether the work represents an advance on the present state of practice in the area	13
Whether the NTRO creator is a substantive university staff member or an adjunct/honorary	16
Whether the NTRO producer was the creator of the work, or a curator/editor of the work?	23
Evidence of academic peer review	23
Whether the NTRO has been critically reviewed in a journal, magazine or book	26
The success of the work according to the criteria of the practice community (i.e. whether is a great piece of art, etc.)	30
Whether the work is unique	33
The scale of the NTRO (e.g. would you score a portfolio of five pieces of work more highly than each of those five pieces submitted as an individual NTRO? A feature film more highly than a short film?)	56%

NTRO: Non-Traditional Research Output.

### Qualitative analysis of criteria with larger numbers of experts nominating ‘It depends’

#### The scale of the NTRO (e

g. would you score a portfolio of five pieces of work more highly than each of those five pieces submitted as an individual NTRO?): 56% say ‘It depends’.

A qualitative analysis of the comments provided by respondents about criteria with larger numbers of experts saying ‘It depends’ suggested that in several cases there was clear disagreement in the cohort about how the criterion should be applied. In these cases, respondents sat along a continuum of approaches, with some at each end and others in the middle.

In relation to the scale of a NTRO, at one end of the continuum sat experts who stated explicitly that scale is not related to academic breakthrough. They made comments such as ‘For example, a ground-breaking essay, short story or poem might be more significant than a full length creative book’ or ‘A very strong single work that causes and communicates a breakthrough in understanding can be of paramount importance’. In making this point, some drew comparison with traditional research outputs, as they noted that ‘you wouldn’t necessarily rank a book more highly because it was a long book, after all’, as they insisted that ‘Scale does not necessarily indicate significance’.

Conversely, at the other end of the continuum, some experts made the opposite point, insisting that ‘Scale, scope or duration plays a role in determining importance or quality . . . Clearly, one painting in a group show is far less impressive than five paintings on the same theme in five different group shows presented as a portfolio’, and ‘it would be hard to say that a single photograph could be assessed higher than a body of works’. The assessors making these points noted that ‘it is more likely that a well-designed portfolio will be able to meet this definition [of significant academic research] than a single piece’, and ‘I believe there is a threshold in regard to minimum “scale” or quantum of research’. For these assessors, ‘poems and other short pieces need to be in portfolios’. As can be seen from the language used, the experts taking these two positions presented their positions as commonsense – ‘Clearly’, ‘you wouldn’t’.

In explaining the need to collate works, some experts used the word ‘minor’. This is an interesting term as its meaning is not settled in the academy: the term could be read literally as meaning ‘smaller’, but there may also exist an implication that ‘minor’ works are those that are less significant. One expert wrote that a ‘portfolio usually gathers a series of minor pieces’, ‘Typically portfolios are used to bundle minor (or weaker) works’ and that because of this ‘I would score individual NTROs (typically those considered of a high enough quality to stand alone) more highly than an NTRO in a portfolio’.

A slightly different perspective – rather than simply addressing scale – is the suggestion that portfolios serve the purpose of showing the emergence of an idea over time. As one expert put it, ‘A portfolio is good for showing the slow emergence of an understanding’. Another commented that ‘Portfolios tend to matter most when the works are very short (e.g. poems or works of microfiction) and/or when there are linked works that all address a particular set of reasonably well-defined preoccupations’. Another expert makes a similar point when saying that

Body of work is a concept that appears in creative arts practice but not in research assessment frameworks. Scale – of individual artwork or of a number of artworks collated for purposes of supervening identity – is relevant to the concept ‘body of work’.

For these respondents, the language of the creative arts – ‘body of work’ is mobilised to explain academic quality of NTROs.

#### Whether the work is unique: 33% say ‘It depends’

This criterion was suggested by the stakeholder group as a criterion that might be used by assessors, but experts felt it was ‘Unclear’. They asked, ‘What is meant by ‘unique’?’ and ‘How is unique defined?’, and insisted that ‘It depends on how you define “unique”’. They also noted that as a criterion it is difficult to judge ‘such claims are contentious and need to be substantiated’. They argued that in terms of operationalising such a criterion, ‘This is almost impossible to gauge’. Another suggested that the criterion would make sense in relation to some genres but not others; ‘it is easier to judge a unique work in a more established field like literature, than it is in popular culture’. Others wrote that ‘Unique is too high as an expectation’; in the same vein, they asked, ‘Is anything unique?’, or simply commented ‘Too hard!’

Other experts drew a distinction between the uniqueness of the *work* and the uniqueness of the *academic research finding*: ‘More important is whether the knowledge that gets generated and communicated is fresh and unique’. From this perspective, ‘The uniqueness of the work, needs to be framed as part of the research questions’ and it ‘Depends . . . whether uniqueness is an important indicator of research success’. As some experts noted, ‘Uniqueness in itself does not guarantee that the work . . . successfully contributes to any particular research environment’. As one expert puts it, ‘In terms of assessment of the NTROs are we asking assessors to be critics or are we asking them to assess research?’ This theme – drawing a distinction between the quality of the work as practice and the quality of the work as academic research – was a recurring and contentious issue in the comments analysed.

#### The success of the work of art according to the criteria of the practice community (i

e. whether it is a great piece of art, etc): 30% say ‘It depends’.

Comments made by experts in relation to this criterion again sat along a continuum, with opposed positions held by numbers of experts at each pole.

On the one hand, some experts believe that ‘A work can “fail” as a creative piece but it can be extremely important if it generates and “illustrates” a breakthrough in understanding’. Similarly, other experts insist that a great work of art is not necessarily academic research: ‘The work first must meet the definitions of research and demonstrate research excellence, and have research significance. This is not the same as peer esteem. A great painting might not contribute new knowledge but just be a great painting’. However, at the other end of the spectrum, some experts insisted strongly that ‘The opinion of a community of practice is the most important element in the assessment of an artwork’, and that ‘No discipline would describe the research as being successful if the outcomes were poor’. Other expert NTRO assessors sat at different places along this continuum, saying, for example, that while practice criteria are not the *only* ones that should be considered, they are among the most important: ‘Important but the audit should be based on the success of the work as a research inquiry not solely on practice-based criteria’; for others, the practice criteria are relevant, but not the most important: ‘This needs to be taken into consideration but, in doing so, it is important to be aware of the different criteria used by practitioners to assess success’. One expert took a slightly different route, pointing out that

given that an NTRO needs to have been made public (for reporting purposes), there is an expectation that it should be able to operate within the public (creative) domain – which is to say, while creative practice as part of a research project may be analogous to lab notes, if such practice results in a reportable NTRO, the work really ought to meet art field values.

Expert assessors in Australia sit right along the continuum in relation to this importance of this criterion.

#### Whether the NTRO has been critically reviewed in a journal, magazine or book; 26% say ‘It depends’

Once again, comments by experts in relation to this criterion sat at along a continuum. Several experts made comments along the lines of: ‘This should be a central guide to quality, worth and research significance’ and ‘A positive critical review in an esteemed journal, magazine or book is one useful way of helping determine its significance’. It is important to note that in this criterion, proposed by the stakeholder group, the review need not be in an academic journal or book – a review in an arts, literature or entertainment magazine would be just as relevant for these assessors. In assigning importance to reviews caveats included ‘the quality of the review outlet’ and whether the review is ‘highly positive’ or ‘negative’. At the other end of the continuum, some experts wrote, ‘There is no correlation between the quality and significance of a NTRO and whether it has been reviewed’. This is an unambiguous refusal of the importance of this criterion. Another notes that

It depends on the . . . rigour of the review with respect to the research contribution . . . obviously it heightens the visibility of a work, but it cannot automatically be considered an analog for research quality (it will most likely be addressing the creative / professional quality).

A critic ‘may not necessarily speak to the new knowledge that has come from the work’ says one expert, while another notes that ‘We will preference a review by a committee of academic peers’ rather than by a non-academic critic.

In each of these contested criteria, we see that there exist in the community of NTRO assessors clear statements supporting their importance, and clear statements rejecting their importance, as well as a variety of positions along each continuum.

## Conclusion

The value of these data comes from showing us how the current community of expert assessors in Australia are engaging with debates about the nature of NTROs as academic research. While there is consensus on four key criteria among Australian experts who are responsible for assessing the quality of NTROs as academic research, the relevance of other criteria remains a topic of evolving discussion. This survey points us to those criteria where there are strong levels of agreement about their importance across the sector:

- Demonstrated familiarity in the research statement with the current state of knowledge in the relevant academic disciplines (very important);
- Demonstrated familiarity in the research statement with the current state of knowledge in the relevant industry (important);
- Evidence that the work has been engaged with by other academic researchers (relevant);
- Whether the NTRO creator is a substantive university staff member or an adjunct/honorary (unimportant).

However, as noted above, this means that there exist 15 criteria identified by stakeholders as possible important in judging the value of NTROs as academic research outputs where there remains disagreements – and in some cases significant disagreements – across the sector about their importance.

Hopefully these data can provide a starting point for the sector to continue discussions and develop more consensus about the importance of such issues. As noted above, the purpose of this project is fundamentally descriptive – to gather data about the way in which experts in Australia are currently making judgements about the value of NTROs as academic research and make it available across the sector for further discussion. It did not set out to take a particular position on these debates, to argue that NTROs should be assessed in a particular way, or to push for particular changes in research policy. Insofar as the article presents an argument, it is this: that research policymakers in Australia considering how to manage the assessment of research outputs going forward – including NTROs – can usefully draw on these data about how research quality is currently being assessed.

## Supplemental Material

Appendix_A_Table_3 – Supplemental material for The criteria used by key decision makers in Australia to judge the academic quality of NTROs Click here for additional data file. Supplemental material, Appendix_A_Table_3 for The criteria used by key decision makers in Australia to judge the academic quality of NTROs by Alan McKee in Media International Australia

Appendix_B – Supplemental material for The criteria used by key decision makers in Australia to judge the academic quality of NTROs Click here for additional data file. Supplemental material, Appendix_B for The criteria used by key decision makers in Australia to judge the academic quality of NTROs by Alan McKee in Media International Australia

Table_4_Appendix_C – Supplemental material for The criteria used by key decision makers in Australia to judge the academic quality of NTROs Click here for additional data file. Supplemental material, Table_4_Appendix_C for The criteria used by key decision makers in Australia to judge the academic quality of NTROs by Alan McKee in Media International Australia
