# Supplementary material for: The criteria used by key decision makers in Australia to judge the academic quality of NTROs
Source: Media Int Aust. 2020 Nov;177(1):165–75. doi: 10.1177/1329878X20921565 (PMC8280536; doi:10.1177/1329878X20921565)
Supplement: Appendix_A_Table_3 – Supplemental material for The criteria used by key decision makers in Australia to judge the academic quality of NTROs [file Appendix_A_Table_3.pdf]

Table 3: Appendix A: List of key NTRO decision makers in Australia approached to complete the survey

Barwick, Linda
Bennett, Jill
Blythe ,Richard
Brien, Donna Lee
Brophy, Kevin
Carlin, David
Del Favero, Dennis
Edmonds, Ernest
Ellison, Liz
Gibson, Ross
Gough, Paul
Groth, Helen
Gwilt, Ian
Harley, Ross
Hetherington, Paul
Holmes, Jonathan
Hope, Cat
Keating, Meg
Kerrigan, Susan
Koehne, Graeme
Kroll, Jeri
Lester, Libby

Lidberg, Johan
Marsh, Kathy
McAuliffe, Chris
Meyrick, Julian
Millard, Kathryn
Miller, Sarah
Muecke, Stephen
Richards, Ian
Sierra, Marie
Tait, Peta
Toltz, Joseph
Vincs, Kim
Webb, Jen
Wise, Kit
Woodrow, Ross
