# Supplementary material for: The criteria used by key decision makers in Australia to judge the academic quality of NTROs
Source: Media Int Aust. 2020 Nov;177(1):165–75. doi: 10.1177/1329878X20921565 (PMC8280536; doi:10.1177/1329878X20921565)
Supplement: Appendix_B – Supplemental material for The criteria used by key decision makers in Australia to judge the academic quality of NTROs [file Appendix_B.pdf]

Appendix B: Survey instrument

1. In which of the following Fields of Research do you have expertise in assessing NTROs?

- 1201 Architecture
- 1203 Design Practice and Management
- 1901 Art Theory and Criticism
- 1902 Film, Television and Digital Media
- 1903 Journalism and Professional Writing
- 1904 Performing Arts and Creative Writing
- 1905 Visual Arts and Crafts
- 2002 Cultural Studies
- 2102 Curatorial and Related Studies
- 2103 Historical Studies
- Other (please specify)

In assessing the quality of a NTRO as academic research, how important is each of the following criteria on a scale from Very unimportant, to Very important?

2. The success of the work according to the criteria of the practice community? (ie, whether it is a great piece of art, successful piece of journalism, good design work, etc)

- Very unimportant
- Unimportant

- Relevant
- Important
- Very important
- It depends (please explain)

Comments: ____

3. The scale of the NTRO (eg, would you score a portfolio of five pieces of work more highly than each of those five pieces submitted as an individual NTRO? A feature film more highly that a short film? etc)

4. The presence of a strong academic research question in the research statement and/or the work itself

5. The rigour of the program of academic research as demonstrated in the research statement and/or the work itself

6. Whether the NTRO has been critically reviewed in a journal, magazine or book

7. The standing as practitioners of other creators whose outputs are presented in the same context (eg, a group show of artists, the company who perform a composition, etc)

8. The standing as academic researchers of other creators whose outputs are presented in the same context (eg, a group show of artists, the company who perform a composition, etc)

9. Whether the work is high culture or popular culture (eg, a literary novel vs a Harlequin Mills and Boon; an experimental short film vs an episode of Home and Away, a news item on the ABC versus a segment of *Today Tonight*, etc)

10. Demonstrated familiarity in the research statement with the current state of knowledge in the relevant academic discipline(s)

11. Demonstrated familiarity in the research statement with the current state of knowledge in the relevant industry

12. Whether the work represents an advance on the present state of practice in the area

13. Whether the work is unique

14. Evidence that the work has been engaged with by other academic researchers (including invitations to present academic keynotes, etc)

15. Whether the work is practice-based, practice-led, or uses practice to report on data generated through a traditional research method (such as a historical novel for example)

16. Whether the NTRO creator is a substantive university staff member or an adjunct/honorary

17. Whether the work has won or been shortlisted for non-academic awards

18. Evidence of industry/community engagement

19. Whether the NTRO producer was creator of the work, or a curator/editor of the work?

20. Evidence of peer review

21. Have you assessed the quality of a dataset as a NTRO?

- Yes
- No

22. If yes, what criteria did you use to assess its academic quality?

Comments: ____

23. Do you have any other comments about the way you assess the quality of a NTRO as a piece of academic research?

Comments: ____
