# Supplementary material for: The criteria used by key decision makers in Australia to judge the academic quality of NTROs
Source: Media Int Aust. 2020 Nov;177(1):165–75. doi: 10.1177/1329878X20921565 (PMC8280536; doi:10.1177/1329878X20921565)
Supplement: Table_4_Appendix_C – Supplemental material for The criteria used by key decision makers in Australia to judge the academic quality of NTROs [file Table_4_Appendix_C.pdf]

Table 4: Appendix C: Statistical analysis at FoR level

Responded to 1203				
Ranked by Adjusted Std Devn				
Ques No.	Aver age	Adj SD	Agree ment	Num Resp
5	5.0	0.00	Unan	2
8	3.0	0.00	Unan	2
10	5.0	0.00	Unan	2
11	5.0	0.00	Unan	2
20	4.0	0.00	Unan	2
2	4.5	0.50	Strong	2
6	3.5	0.50	Strong	2
12	4.5	0.50	Strong	2
14	3.5	0.50	Strong	2
16	2.5	0.50	Strong	2
17	3.5	0.50	Strong	2
18	4.5	0.50	Strong	2
7	3.5	3.00	None	2
9	2.5	3.00	None	2
15	2.5	3.00	None	2
3	4.0		Only 1	1
4	5.0		Only 1	1
13	5.0		Only 1	1
19	2.0		Only 1	1

Responded to 1203

Responded to 1901				
Ranked by Adjusted Std Devn				
Ques No.	Aver age	Adj SD	Agree ment	Num Resp
10	4.8	0.42	Strong	9
6	3.3	0.43	Strong	8
3	4.3	0.47	Strong	3
14	3.4	0.50	Strong	9
5	4.6	0.77	Strong	9
17	3.5	0.81	Strong	8
8	3.3	0.82	Strong	7
16	2.3	0.82	Strong	7
11	4.1	1.04	Fair	8
12	4.1	1.04	Fair	8
18	3.7	1.05	Fair	9
4	4.3	1.11	Fair	8
20	3.1	1.15	Fair	7
2	4.6	1.22	Mild	7
19	3.4	1.47	Weak	7
15	3.0	1.49	Weak	10
13	3.8	1.63	Weak	5
7	3.7	2.08	None	9
9	3.0	3.06	None	7

Responded to 1901

Responded to 1902				
Ranked by Adjusted Std Devn				
Ques No.	Aver age	Adj SD	Agree ment	Num Resp
3	4.2	0.40	Strong	5
14	3.7	0.47	Strong	9
6	3.4	0.49	Strong	5
11	4.3	0.75	Strong	9
17	3.8	0.76	Strong	8
2	4.5	0.81	Strong	8
16	2.5	0.92	Strong	6
18	4.0	0.94	Strong	8
12	4.2	1.01	Fair	9
15	3.3	1.29	Mild	10
10	4.5	1.34	Mild	10
8	2.6	1.78	Weak	8
20	3.7	1.80	Weak	7
5	3.9	1.89	None	10
4	3.8	1.99	None	9
7	3.5	2.24	None	8
13	3.1	2.37	None	7
9	3.0	2.83	None	8
19	3.3	3.23	None	7

Responded to 1902

Responded to 1903				
Ranked by Adjusted Std Devn				
Ques No.	Aver age	Adj SD	Agree ment	Num Resp
4	4.7	0.47	Strong	3
10	4.3	0.47	Strong	3
11	4.3	0.47	Strong	3
15	3.7	0.47	Strong	3
16	1.3	0.47	Strong	3
19	3.3	0.47	Strong	3
7	1.5	0.50	Strong	2
8	2.0	1.22	Mild	3
17	2.3	1.41	Mild	3
18	3.3	1.87	None	3
6	2.0	2.00	None	2
13	3.0	2.00	None	2
9	2.3	2.83	None	3
3	3.5	3.00	None	2
12	3.5	3.00	None	2
5	3.7	3.74	None	3
14	2.7	3.74	None	3
20	2.7	3.74	None	3
2	4.0		Only 1	1

Responded to 1903

Responded to 1904				
Ranked by Adjusted Std Devn				
Ques No.	Aver age	Adj SD	Agree ment	Num Resp
3	3.3	0.47	Strong	6
14	3.7	0.81	Strong	15
2	4.4	1.00	Fair	10
16	1.8	1.05	Fair	13
18	3.8	1.14	Fair	15
10	4.5	1.17	Fair	17
8	2.3	1.19	Fair	15
4	4.3	1.24	Mild	15
11	4.2	1.28	Mild	16
5	4.2	1.29	Mild	17
15	2.9	1.33	Mild	17
6	3.7	1.45	Weak	11
17	3.6	1.47	Weak	14
12	4.0	1.72	Weak	15
7	3.3	1.87	None	15
20	3.2	1.93	None	13
13	3.3	2.23	None	13
9	2.7	2.25	None	15
19	3.5	2.26	None	13

Responded to 1904

Responded to 1905				
Ranked by Adjusted Std Devn				
Ques No.	Aver age	Adj SD	Agree ment	Num Resp
6	3.1	0.33	Strong	8
10	4.8	0.42	Strong	9
14	3.3	0.47	Strong	9
5	4.7	0.75	Strong	9
17	3.3	0.75	Strong	9
11	4.3	0.76	Strong	8
16	2.3	1.05	Fair	9
4	4.3	1.06	Fair	9
13	3.2	1.08	Fair	6
12	3.9	1.12	Fair	9
2	4.4	1.13	Fair	8
3	4.0	1.22	Mild	3
19	3.6	1.26	Mild	7
15	2.7	1.29	Mild	10
18	3.6	1.31	Mild	10
20	3.4	1.48	Weak	8
9	2.1	1.69	Weak	8
8	3.3	1.92	None	8
7	3.1	2.60	None	10

Responded to 1905

Responded to 2002				
Ranked by Adjusted Std Devn				
Ques No.	Aver age	Adj SD	Agree ment	Num Resp
4	5.0	0.00	Unan	2
5	5.0	0.00	Unan	2
10	5.0	0.00	Unan	2
11	5.0	0.00	Unan	2
12	5.0	0.00	Unan	2
14	4.0	0.00	Unan	2
2	4.5	0.50	Strong	2
7	4.5	0.50	Strong	2
8	3.5	0.50	Strong	2
17	4.5	0.50	Strong	2
18	3.5	0.50	Strong	2
9	3.5	3.00	None	2
16	2.5	3.00	None	2
19	3.5	3.00	None	2
6	4.0		Only 1	1
13	5.0		Only 1	1
15	4.0		Only 1	1
20	3.0		Only 1	1
3			0 Resp	-

◀ ◀ ◀ Responded to 2002

Responded to 2102				
Ranked by Adjusted Std Devn				
Ques No.	Aver age	Adj SD	Agree ment	Num Resp
4	5.0	0.00	Unan	2
5	5.0	0.00	Unan	2
6	3.0	0.00	Unan	2
10	5.0	0.00	Unan	2
12	5.0	0.00	Unan	2
14	3.0	0.00	Unan	2
16	2.0	0.00	Unan	2
17	3.0	0.00	Unan	2
18	3.0	0.00	Unan	2
9	2.5	0.50	Strong	2
7	3.0	2.00	None	2
8	3.0	2.00	None	2
15	3.0	2.00	None	2
2	3.5	3.00	None	2
11	3.0		Only 1	1
13	3.0		Only 1	1
19	3.0		Only 1	1
20	4.0		Only 1	1
3			0 Resp	-

◀ ◀ ◀ Responded to 2102
